# Supraorbital Bone Shaving (S.O.S Procedure) in Restoration of Upper Lateral Convexity in Sunken Eyes

**DOI:** 10.1007/s00266-024-04194-9

**Published:** 2024-06-28

**Authors:** Ufuk Askeroglu, Fatih Ceran, Ozgur Pilanci

**Affiliations:** 1 Istanbul, Turkey; 2 Department of Plastic, Reconstructive and Aesthetic Surgery, Faculty of Medicine, Biruni University, Beşyol, Eski Londra Asfaltı No:10, 34295 Küçükçekmece, Istanbul, Turkey

**Keywords:** Eyes, Supraorbital, Shaving, Sunken, Reconstruction, Aesthetic, Upper lateral convexity, Restoration

## Abstract

**Background:**

Sunken eyes have become a most important target of periorbital area aesthetics. Throughout history, the aesthetics of the periorbital region have been emphasized, and various surgical techniques related to this region have been described. Most of these techniques provide only soft tissue solutions; therefore, additional surgical interventions may be required. The aim of our study was to introduce an endoscopic supraorbital shaving (SOS) technique for the treatment of individuals with sunken eyes.

**Methods:**

Between 2020 and 2021, 34 patients (30 females, 4 males; mean age 36.2 years) with sunken eyes were treated with our described technique. All patients underwent an endoscopic SOS procedure under general anesthesia.

**Results:**

A total of 34 patients (30 women and four men), aged 23–59 years old (mean = 36.2 years), underwent the endoscopic SOS procedure. The mean follow-up period was 13 months (range: 12–16 months). Postoperatively, significant improvement in lateral convexity was achieved in all patients. Physical examinations performed at the control visits revealed no functional problems in any patients and no visible or palpable irregularities or contour deformities. No complications were encountered regarding the SOS procedure.

**Conclusions:**

The technique described here provides significant improvement in lateral convexity compared to other techniques used in patients with sunken eyes. No additional eyelid intervention is needed. Unlike the techniques previously described in the literature, intervention is made in the bone structure, thereby providing more accurate results.

**Level of Evidence V:**

This journal requires that authors assign a level of evidence to each article. For a full description of these Evidence-Based Medicine ratings, please refer to the Table of Contents or the online Instructions to Authors www.springer.com/00266.

**Supplementary Information:**

The online version contains supplementary material available at 10.1007/s00266-024-04194-9.

## Introduction

As human life expectancy increases, having an aesthetically pleasing and youthful face becomes an inevitable desire at any age for many people. Focusing on the aesthetic trends, one of the most important points in the upper third region of the face is the aesthetic features of the periorbital region. An aesthetically perfect eyelid can be defined by the fullness in the lateral convexity of the upper eyelid from the eyebrow to the eyelid crease and a sharp and well-defined eyelid crease [1, 2]. Upper eyelids with sunken, hollow, skeletonized orbits can make the person look older, tired, and skinny [3]. In people with sunken eyelids, depth is more evident in the central part of the upper eyelid, and a loss of lateral convexity is apparent [4, 5].

Although sunken eyelids are generally seen in the elderly population, they can be structural in patients between the ages of 20 and 50 years. Trauma, mostly resulting from traffic accidents, can cause depressions in both the upper and lower eyelids. Another factor that should be emphasized is the sunken eyelid deformity that occurs iatrogenically after blepharoplasty operations due to fat pad removal [1–5].

The available literature mostly describes the solutions found for elderly and Asian populations and was most were interventions on soft tissue. Autologous fat grafting, dermofat grafting, fascia-fat grafting, orbicularis muscle imbrication, orbital fat repositioning, and dermal filler injection are among some of the treatments performed to treat this deformity [5–11]. However, the thin structure of the skin of the upper eyelid may prevent full addressing of this deformity. Another important detail that is overlooked in these operations is that the lateral convexity is not completely corrected. In these operations, the autologous fat graft may undergo absorption and misplacement, contour irregularities may occur, and multiple additional procedures may be necessary.

Our aim in this manuscript was to define a new endoscopically designed approach, which we call supraorbital shaving (SOS). This SOS procedure restores upper orbital convexity, but its use of bone shaping distinguishes it from previously described methods based on soft tissue reconstruction.

## Materials and Methods

### Patients

Between February 2020 and February 2021, a total of 34 patients (30 females, 4 males) presenting prominent supraorbital rim were treated. Restoration of lateral orbital convexity with this particular technique was performed as an adjunctive procedure in patients seeking face rejuvenation procedures, such as face lifts, Bella eyes (Bella eyes are an innovative surgical technique that combines endoscopic dynamic canthopexy and lateral brow lift), Trinity lift (this is a suspension and fixation method for an endoscopic midface lift with the aid of a percutaneous needle and to present the outcomes of this particular technique), and eyelid ptosis correction. Preoperative and postoperative photos taken at the 12-month visit were compared.

## Surgical Technique

A total of 20 mL of infiltration fluid consisting of 0.5% bupivacaine plus epinephrine (diluted 1:100000) were administered under general anesthesia to the zone of dissection at the temple and periorbital region, especially the caudal and cranial aspects of the supraorbital bony rim. A single 2.5 cm incision was made behind the hairline and parallel to the hair follicles, approximately 8 cm laterally to the midline. After skin incision, 2 cm of limited subcutaneous dissection was performed caudally, proceeding with the subfascial and subperiosteal planes and exposing the supraorbital bone under endoscopic guidance. Special care was taken to avoid any damage to the supraorbital bundle. A wide exposure was made of the supraorbital bone lateral to the bundle and continuing laterally to the lateral orbital rim. Release of the inner aspects of the lateral and supraorbital periosteum was checked with a fine-edged curved bone elevator.

No marking was done for orbital bone shaving; however, in all cases, the excess bony prominence was easily detected as the endoscope was advanced caudally from the scene, and this zone was double checked with the aid of fingertip palpation. A piezo device (Woodpecker Co.) was used for bone shaping. One curved-edge attachment and a smaller straight-tipped piezo attachment were utilized for this purpose. The contour of the orbital rim was gradually checked from outside while the flap was retracted to the desired vector to the temple zone.

## Results

**Fig. 1:**
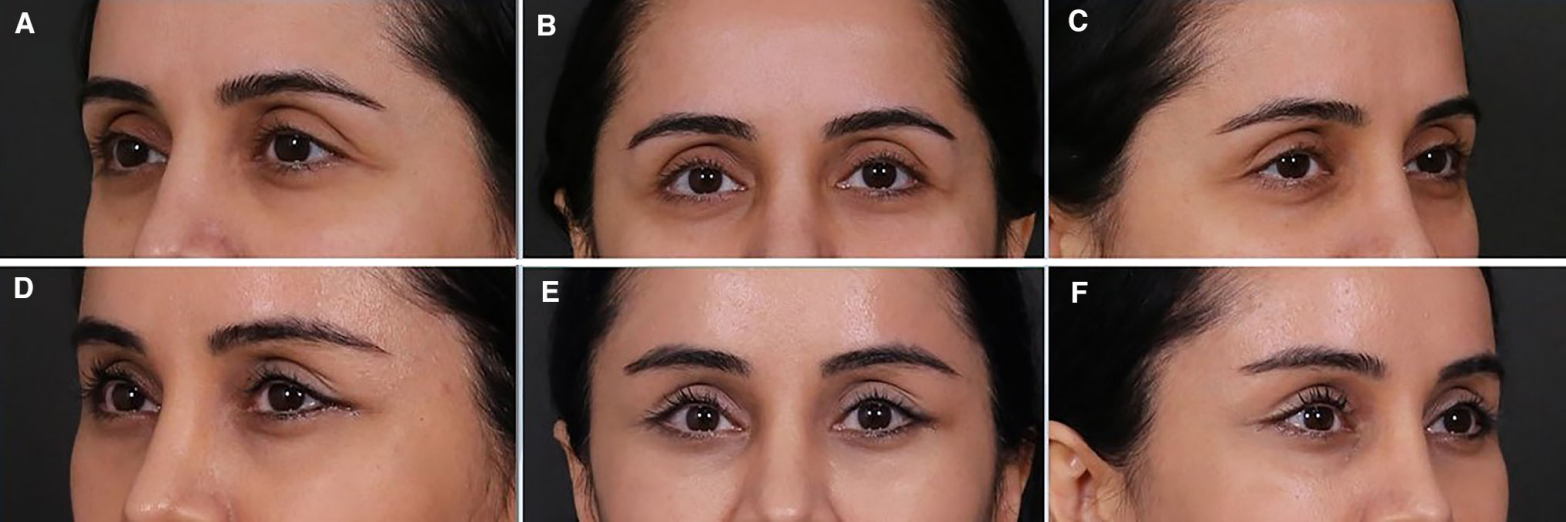
Preoperative (**A**, **B**, **C**) and postoperative view (**D**, **E**, **F**) of patient undergone SOS procedure combined with Bella eyes and Trinity lift

**Fig. 2:**
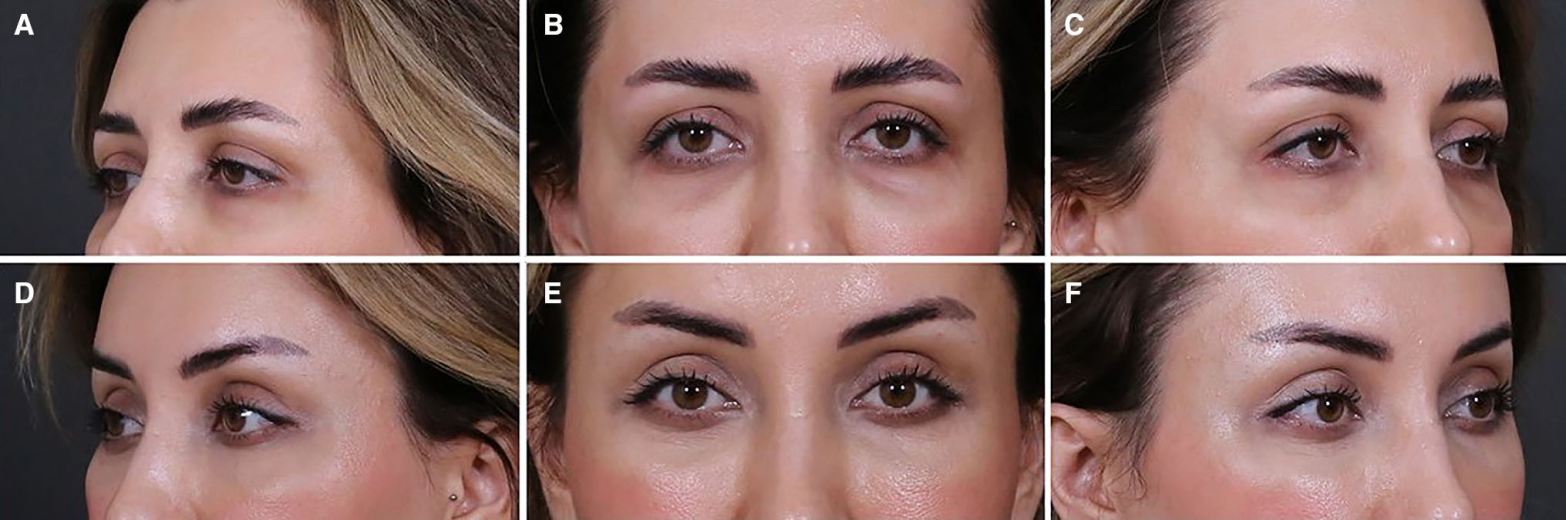
Preoperative (**A**, **B**, **C**) and postoperative view (**D**, **E**, **F**) of patient undergone SOS procedure adjuncted to Bella eyes, Trinity lift, ptosis, brow lift, and fat injection

**Fig. 3:**
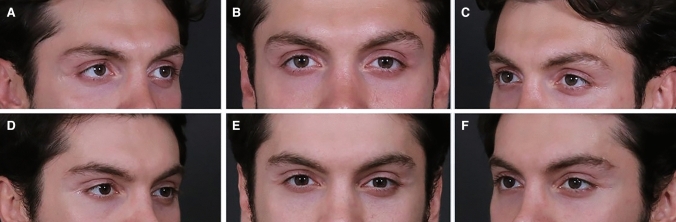
Preoperative (**A**, **B**, **C**) and postoperative view (**D**, **E**, **F**) of patient undergone SOS procedure combined with Bella eyes

In total, 34 patients undergoing SOS procedures were evaluated. The patients were followed for an average of 12 months (Table 1). Pre- and postoperative photograph comparisons displayed a considerable improvement in the upper lateral eyelid convexity, resulting in a youthful appearance. No visible or palpable irregularities or contour deformities were evident. No complications were encountered regarding the SOS procedure. All the results remained stable throughout the follow-up period. Patient satisfaction was good to excellent in all cases (Figs. 1, 2 and 3).

**Table 1:** Characteristics of the patients and the interventions underwent

Patient	Age	Gender	Adjunctive procedures
1	25	M	Brow lift, ptosis, Bella eyes
2	33	F	Bella eyes, Trinity lift, brow lift, fat graft
3	29	F	Bella eyes, blepharoplasty, fat grafting
4	36	F	Bella eyes, Trinity lift, brow lift
5	36	F	Bella eyes, ptosis, Trinity lift, lip lift, fat grafting
6	30	M	Bella eyes and ptosis
7	39	F	Bella eyes and Trinity lift
8	47	F	Trinity lift, brow lift, fat grefting
9	34	F	Bella eyes and Trinity lift
10	38	F	Bella eyes, Trinity lift, ptosis, brow lift
11	36	F	Bella eyes, Trinity lift, ptosis
12	35	F	Trinity lift, Brow lift
13	41	F	Bella eyes, Trinity lift, Lip lift
14	45	F	Bella eyes, Trinity lift, ptosis
15	27	F	Bella eyes, Trinity lift, brow lift
16	32	F	Bella eyes, Trinity lift, ptosis
17	24	F	Bella eyes
18	27	F	Bella eyes
19	37	F	Brow lift and Trinity lift
20	26	M	Brow lift
21	23	F	Bella eyes
22	39	F	Bella eyes, Trinity lift, brow lift
23	34	F	Trinity lift and Bella eyes
24	38	F	Brow lift, Trinity lift, Bella eyes
25	42	F	Trinity lift, ptosis, Bella eyes
26	59	F	Bella eyes, Trinity lift, brow lift
27	43	F	Trinity lift, ptosis, Bella eyes
28	42	F	Trinity lift
29	42	F	Trinity lift and ptosis
30	44	F	Bella eyes and Trinity lift
31	34	M	Bella eyes and Trinity lift
32	31	F	Bella eyes and brow lift
33	39	F	Bella eyes and Trinity lift
34	47	F	Face lift

## Discussion

Periorbital aging manifests as declining eyebrows, a shorter distance between the eyebrows and eyelashes, and wrinkling and folding of the upper eyelid skin, as well as excessive skin of the upper eyelid [1, 2]. Since all the patients included in our study underwent supraorbital bone shaving combined with temporal and brow lift, rejuvenations of all these regions were achieved simultaneously, providing a harmonious improvement.

Gulyas published a study on his technique that involves resection of the conservative medial fat compartment and relocation of the diced fat into an imbricated orbicularis oculus muscle to increase the convexity of the upper eyelid [2]. Coleman and Fagien published a micrograft technique in the preorbital region [1, 9]. Although these techniques were effective in many patients, the likelihood of these fat grafts to be permanent or not absorbed changes, either from patient to patient or depending on the technique applied by surgeons. Neither fat graft reabsorption nor its permanence can be easily predicted. These techniques have produced truly successful results. Hamra popularized the fat pad sliding technique to correct the tear-through deformity and volume loss previously described by Loeb [10, 11]. Sozer et al. achieved very successful results for restoration of the convexity at the upper eyelid by transferring the orbital branches of the angular and ophthalmic artery to the lateral compartment using pedicles to the upper eyelid central compartment or fat pad [5]. However, in his study, Sozer emphasized this technique was applicable only for those patients who had excess or herniated upper central fat pads. In particular, this technique was not effective in patients who had thin subcutaneous skin and insufficient fat pads. The SOS technique, by contrast, offers a possibility for intervention in a broader spectrum of patients, since it is not based on the volume of available fat pad.

Another aspect of our study was that the orbital prominence was prevented from becoming even more apparent/distinct during the brow lift, particularly in patients with lower eyebrows. We believe that the SOS technique allows a smoother transition between the lateral orbital zone and the temporal region. Another important point that arose from our study was that no incisions are made on the upper eyelid during this treatment, and no layers of the eye or its anatomical integrity are violated. This procedure can be performed using a single entry point in the temporal hair and in conjunction with an endoscopic brow lift. We believe that the results can be more precise and predictable, since we can check the upper eyelid contour with the aid of lifting the brow in the desired vector.

This report has some limitations, particularly the number of patients evaluated, and the longevity of the results may not be sufficient. In addition, the surgical outcome was primarily subjective and photo based, which may represent additional drawbacks of our study.

## Conclusion

We found the SOS procedure to be a very effective method for restoring the convexity in the outer corner of the upper eyelid. It does not require any extra eyelid intervention and can be used safely, especially in patients undergoing an endoscopic brow lift and temporal lift. We believe that the supraorbital bone shaving part of the study is also valuable, as this has not been described before in the literature. This study is the first to describe an intervention using bone and hard tissue, rather than the traditional soft tissue interventions made to provide eyelid convexity.

## Supplementary Information

Below is the link to the electronic supplementary material.

Video clearly showing shaving the orbital rim and ensuring lateral convexity with the help of a piezzo device. (MOV 51560 kb)
